# Modulation of Intestinal Energy Metabolism and Microbial Profiles by Dietary Starch Characteristics Under EGCG Supplementation in Broiler Chickens

**DOI:** 10.3390/ani16152445

**Published:** 2026-08-06

**Authors:** Wanqin Liu, Ruiyang Zhang, Yanli Zhu, Kai Liang, Dafei Yin

**Affiliations:** 1 College of Animal Science and Veterinary Medicine, Shenyang Agricultural University, Shenyang 110866, China; 2 College of Animal Husbandry and Veterinary Medicine, Liaoning Agricultural Vocational and Technical College, Yingkou 115009, China

**Keywords:** starch, EGCG, intestinal energy metabolism, microbial metabolism, broiler

## Abstract

This study evaluated the effects of dietary starch characteristics on intestinal energy metabolism and microbial profiles in Epigallocatechin gallate (EGCG)-supplemented broiler chickens. A total of 300 Arbor Acres male broilers were assigned to five dietary treatments consisting of a corn–soybean basal diet (NC), EGCG supplementation (500 mg/kg) (PC), or diets in which 20% corn was substituted with purified corn (CS), cassava (TS), or pea starch (PS) in the presence of EGCG (500 mg/kg). Growth performance, starch digestion, intestinal energy status, cecal microbiota, and metabolomic profiles were evaluated. The results showed that broilers fed the corn starch-containing diet under EGCG supplementation showed improved average daily gain and feed conversion ratio (*p* < 0.05). This treatment also increased intestinal ATP and cAMP concentrations while reducing AMP and the AMP/ATP ratio, accompanied by enhanced activities of Na^+^-K^+^-ATPase, citrate synthase, and pyruvate dehydrogenase. Cecal microbiota analysis showed that *Lactobacillus* abundance was increased in the CS group, whereas *Barnesiella* was enriched in the PS group. Differential metabolites associated with energy metabolism, including lipoic acid, palmitoylcarnitine, and crotonic acid, were significantly changed among treatments. In conclusion, dietary starch characteristics influenced intestinal energy metabolism-related indicators, microbial profiles, and metabolite patterns in broilers receiving EGCG supplementation.

## Simple Summary

Efficient intestinal energy utilization depends not only on dietary starch content, but also on the dynamics of glucose release and utilization. The present study demonstrates that Epigallocatechin gallate (EGCG), a major polyphenol in green tea, modulated intestinal energy metabolism in a starch source-dependent manner, thereby altering microbial composition and metabolic responses in broilers. Among the different starch sources, a corn starch-containing diet under EGCG supplementation provided a more stable intestinal energy status, which was associated with improved growth performance and enrichment of beneficial microbiota. These findings prove the importance of synchronizing starch digestion kinetics with intestinal metabolic demand and provide a potential nutritional strategy for improving energy efficiency and gut functionality in poultry production.

## 1. Introduction

Efficient utilization of dietary energy is essential for sustainable poultry production, with starch serving as the primary carbohydrate source in broiler diets. The digestion of starch in the small intestine generates glucose, which acts as a key substrate for cellular energy metabolism [1,2]. Importantly, the rate and pattern of glucose release, rather than the total amount of starch, play a critical role in determining intestinal energy supply, metabolic efficiency, and growth performance [3,4,5].

Different starch sources exhibit distinct physicochemical properties and digestion kinetics, resulting in different patterns of glucose release. Cassava starch, characterized by a low amylose-to-amylopectin ratio, is rapidly digested and leads to rapid glucose release, which induces extremely high increases in luminal glucose concentrations and exceeds the absorptive capacity of enterocytes and disrupts metabolic homeostasis [6]. In contrast, pea starch, with a higher amylose content, is digested more slowly and provides a sustained glucose release [7,8], but their lower digestibility may limit overall energy availability [9]. Corn starch shows relatively high digestibility and intermediate glucose release characteristics, which could potentially reduce amino acid oxidation for energy supply [10,11]. These contrasting characteristics underscore a key challenge in poultry nutrition, namely, how to match glucose release kinetics with intestinal energy demand in order to improve metabolic efficiency.

Epigallocatechin gallate (EGCG), a major polyphenol in green tea, has been reported to influence starch digestion and glucose metabolism. EGCG can bind to starch molecules and digestive enzymes, thereby modifying enzymatic hydrolysis and glucose release [12,13,14]. Additionally, EGCG has been shown to enhance glucose absorption by upregulating glucose transporters, such as SGLT1 and GLUT2, thereby improving glucose uptake efficiency [15]. Furthermore, EGCG supports overall energy metabolism by preserving ATP production and reducing oxidative stress [16,17,18]. Lin et al. [19] also reported that the regulatory mechanisms of energy metabolism involve interactions between dietary polyphenols and the intestinal microbiota, wherein polyphenols modulate the composition and activity of commensal bacteria, thereby exerting beneficial effects on the host. However, whether starch sources with different digestion characteristics produce distinct intestinal metabolic responses under EGCG supplementation remains unclear.

According to these considerations, it was hypothesized that dietary starch characteristics may influence glucose release and intestinal energy status under EGCG supplementation, accompanied by alterations in microbial composition and metabolic profiles. Therefore, the present study was conducted to evaluate the effects of dietary starch characteristics under EGCG supplementation on starch digestion, digestive enzyme activities, glucose transport, intestinal energy metabolism, microbiota composition, and metabolite profiles in broiler chickens. The findings are expected to provide further insight into nutritional strategies for improving energy utilization and metabolic efficiency in poultry production.

## 2. Materials and Methods

### 2.1. Animal Ethics

The animal experiment was approved by the Animal Welfare and Ethical Committee of Shenyang Agricultural University (Approval No. 23031204).

### 2.2. Materials

Epigallocatechin gallate (EGCG, purity ≥ 98%) was obtained from Xi’an Tongze Biotech Co., Ltd. (Xi’an, China). Purified corn starch, cassava starch, and pea starch were purchased from Ingredion China Co., Ltd. (Shanghai, China).

### 2.3. Experimental Design and Dietary Treatments

A total of 300 male Arbor Acres broilers were used in this study. Before dietary intervention, all birds were fed a commercial starter diet from hatch to 13 d of age. At 14 d, broilers with similar initial body weight were randomly allocated to five dietary treatments, with six replicate cages per treatment and 10 broilers per cage. The experimental treatments consisted of a basal corn–soybean meal diet without EGCG supplementation (NC), the basal diet supplemented with 500 mg/kg EGCG (PC), and three additional diets in which 20% of dietary corn was substituted with purified corn starch (CS), cassava starch (TS), or pea starch (PS), respectively, in the presence of 500 mg/kg EGCG. Diet formulations were prepared according to the Chinese Feeding Standard for Chickens (NY/T 33-2004) [20] (Table 1). Feed and water were provided ad libitum throughout the experimental period.

Broilers were reared under environmentally controlled conditions. Ambient temperature was maintained at 33 °C during the first week and gradually decreased to 26 °C by 21 d of age. A lighting schedule of 23 h light and 1 h darkness was applied during the trial.

### 2.4. Growth Performance and Postprandial Glucose Response

Body weight and feed consumption were recorded on a replicate basis at 35 and 42 d following a 12 h fasting period. Average daily gain (ADG), average daily feed intake (ADFI), and feed conversion ratio (FCR) were subsequently calculated.

To evaluate postprandial glucose dynamics, six birds from each treatment with body weight close to the replicate mean were selected at 28 d of age. Following feed deprivation for 12 h, blood samples were collected from the wing vein before feeding and at 1, 2, 3, and 4 h after refeeding. Blood glucose concentration was determined immediately using a portable glucometer (ACCU-CHEK Performa, Roche Diagnostics, Germany).

### 2.5. Sample Collection

At 42 d, one broiler with a body weight close to the average value was randomly selected from each replicate. Jejunal and ileal digesta were collected, rapidly frozen, and stored at −80 °C for determination of starch digestibility [21,22]. Carefully incise the jejunum and ileum lengthwise, rinse them gently with a 0.9% sodium chloride solution, and use clean slides to gently scrape samples from the mucosa of the jejunum and ileum, respectively [23]. Collect these samples in 1.5-milliliter sterile tubes and freeze them in liquid nitrogen and preserve at −80 °C for biochemical and molecular analyses. Cecal digesta samples were aseptically collected and stored at −80 °C prior to microbiota sequencing and metabolomic analysis. Additionally, mid-jejunum and mid-ileum sections (0.5 cm long) were also collected in RNase-free microcentrifuge tubes and frozen in liquid nitrogen and stored at −80 °C for gene expression analysis.

### 2.6. Intestinal Starch Utilization and Enzyme Activities

For starch digestibility determination, freeze-dried digesta and diet samples were finely ground prior to analysis. Starch concentration was quantified using commercial assay kits (Solarbio, Beijing, China, No. BC0615), whereas acid-insoluble ash (AIA) was used as an internal marker according to Siriwan et al. [24]. Apparent digestibility of starch was calculated by the following equation:

Starch Digestibility (%) = 100 − (AIA _diet_/AIA _digesta_× Starch _digesta_/Starch _diet_ × 100)

Jejunal mucosal samples were used for the determination of α-amylase (EC 3.2.1.1), sucrase (EC 3.2.1.48), and maltase (EC 3.2.1.20) activities. Briefly, 0.2 g of mucosal tissue was mixed with chilled saline solution and homogenized at a 1:6 (*w*/*v*) ratio using a tissue homogenizer (JIUPIN, Wuxi, China). The homogenates were subsequently used for enzymatic analysis with commercial assay kits (C016, A082-2-1, and A082-3-1; Nanjing Jiancheng Bioengineering Institute, Nanjing, China) according to the manufacturer’s recommendations.

### 2.7. Evaluation of Intestinal Energy Homeostasis

Jejunal and ileal mucosal tissues were collected for the evaluation of intestinal energy-related metabolites and enzyme activities. The concentrations of ATP, AMP, and cAMP were quantified using commercial assay kits (JM-09309C1, JM-09482C1, and JM-09487C1; Jingmei Biotechnology, Yancheng, China), and the AMP/ATP ratio was subsequently calculated.

The activities of Na^+^/K^+^-ATPase, citrate synthase (EC 2.3.3.1, Cs), and pyruvate dehydrogenase (EC 1.2.4.1, PDH) in intestinal mucosa were measured using corresponding commercial kits (JM-05008C1, JM-09491C1, and JM-09496C1; Jingmei Biotechnology, Yancheng, China) according to the manufacturer’s protocols.

### 2.8. Expression of Genes Related to Glucose Transport and Energy Metabolism

RNA extraction procedure was followed by Xi et al. [25] with appropriate modifications. Total RNA was isolated from jejunal and ileal tissues using TRIzol reagent (ThermoFisher Scientific, OH, USA). The extracted RNA was reverse-transcribed into cDNA using a commercial reverse transcription kit (RR047A, Takara, Beijing, China). Quantitative real-time PCR analysis was subsequently conducted with a SYBR Green PCR kit (RR420A, Takara, Beijing, China) on an Applied Biosystems 7500 Real-Time PCR System (Foster City, CA, USA). Primer sequences used in the present study were synthesized by Sangon Biotech (Shanghai, China) and are listed in Table 2. β-actin was used as the reference gene for normalization. Relative mRNA abundance was calculated using the 2^−ΔΔCt^ method, with expression levels normalized to the PC treatment group. Primer specificity was verified by melting curve analysis, whereas primer amplification efficiencies were not experimentally determined.

### 2.9. DNA Extraction and 16S rRNA Sequencing

Genomic DNA was extracted from frozen cecal contents using the Magnetic Soil and Stool DNA Kit (TianGen, China, No. DP712). The microbial 16S rRNA gene was amplified using specific primers: 341F (5′-CCTAYGGGRBGCASCAG-3′) and 806R (5′-GGACTACNNGGGTATCTAAT-3′), targeting the V3-V4 region. The PCR products were then purified using the Universal DNA Purification Kit (Tiangen, China, No. DP214). Sequencing libraries were prepared with the NEB Next^®^ Ultra™ II DNA Library Prep Kit (New England Biolabs, MA, USA, Catalog No. E7645) following the manufacturer’s instructions. Paired-end sequencing (250 bp) was performed.

Based on the unique barcodes of each sample, the sequencing reads were assigned to the corresponding samples, followed by trimming of barcode and primer sequences. The reads were merged using the FLASH tool (V1.2.1). Raw tags were quality-filtered using Fastp (V0.23.1) to generate high-quality clean tags. The effective tags were compared against the SILVA 16S rRNA reference database (https://www.arbsilva.de/, accessed on 30 October 2022). Denoising was performed using QIIME2 (V2-202006) to obtain Amplicon Sequence Variants (ASVs), and ASVs with less than five occurrences were filtered out. Taxonomic classification was performed using QIIME2 with the Silva database. The 16S rRNA gene amplicon sequencing data generated in this study were submitted to the NCBI Sequence Read Archive (SRA), accession number: “PRJNA1457713”.

### 2.10. Bioinformatics Analysis

Alpha diversity was assessed using the following indices: Chao1, Dominance, Shannon, Simpson, and Pielou’s evenness index (Pielou_e). Beta diversity was assessed using Bray–Curtis dissimilarity, and principal coordinates analysis (PCoA) was performed to visualize differences in microbial community composition among treatments.

Differentially abundant microbial taxa among dietary treatments were identified using linear discriminant effect size analysis (LEfSe software version 1.0). The analysis was performed based on the relative abundance profiles of six biological replicates per treatment. Taxa with a Kruskal–Wallis test *p* < 0.05 and an LDA score > 2.0 were considered differential microbial taxa. [26]. Spearman’s rank correlation analysis was performed to explore the relationship between the microbiota composition and antioxidant capacity, based on the relative abundances at the genus level [27].

### 2.11. Metabolite Extraction, Profiling, and Analysis

The metabolome of cecal digesta from birds was analyzed via the untargeted LC-MS-based metabolomics approach [28]. Briefly, samples were homogenized with methanol and centrifuged at 12,000 rpm for 10 min at 4 °C. The supernatants were collected, vacuum-dried, reconstituted, and filtered through a 0.22 μm membrane before LC-MS/MS analysis. Quality control (QC) samples were prepared by pooling equal aliquots from all samples and injected throughout the analytical sequence to monitor analytical stability. Features with QC CV < 30% were considered reproducible. Procedural blank samples were not included during sample preparation or LC-MS/MS analysis.

Metabolomic profiling was performed using a UHPLC-Q Exactive Orbitrap MS system (Thermo Fisher Scientific, Waltham, MA, USA) with electrospray ionization in both positive and negative modes. Chromatographic separation was achieved using an ACQUITY UPLC HSS T3 column. Raw data were processed using Compound Discoverer 3.1 for peak extraction, alignment, and metabolite annotation based on accurate mass and MS/MS fragmentation patterns. Features with missing values exceeding 50% were removed, and remaining missing values were imputed using half of the minimum detected value. Differential metabolites were screened according to VIP > 1, *p* < 0.05, and fold change ≥ 1.5 or ≤ 0.667. No false discovery rate (FDR) correction was applied during metabolite screening. Metabolic pathway enrichment analysis was conducted using MetaboAnalyst 4.0 based on the KEGG database.

### 2.12. Statistical Analysis

All data were analyzed using SPSS Statistics (Version 26.0, IBM, Armonk, NY, USA). Prior to the analysis of variance, Shapiro–Wilk’s test was performed to check the normality, and Levene’s test was conducted for homogeneity of variance. A one-way analysis of variance (ANOVA) was conducted to compare treatment groups. Duncan’s post hoc test was applied for multiple comparisons when significant differences were detected. Significance was set at *p* < 0.05.

## 3. Results

### 3.1. Growth Performance

Growth performance of broiler chickens receiving the five dietary treatments is presented in Table 3. There was no significant difference in 14 d body weight among all the treatments (*p* > 0.05). However, the PC group significantly decreased 35 d and 42 d body weight compared with the other groups (*p* < 0.02). From 14 to 35 d, birds in the PC group had lower ADG and ADFI and a higher FCR than those in the other groups (*p* < 0.05), whereas no differences were observed among the NC, CS, TS, and PS groups. From 35 to 42 d, dietary treatment did not affect ADG or FCR, but ADFI was lower in the PC group than in the other groups (*p* < 0.05). Over the entire experimental period from 14 to 42 d, the PC group showed lower ADG and ADFI than the NC, CS, TS, and PS groups (*p* < 0.05), while FCR was not affected by dietary treatment.

From 35 to 42 d, ADFI was increased in the CS group compared with the PC treatment (*p* < 0.05). Over the entire period (14–42 d), birds fed the CS diet maintained greater ADG and improved FCR relative to the PC group (*p* < 0.05), whereas TS and PS treatments showed intermediate responses.

### 3.2. Postprandial Blood Glucose Response

**Figure 1:**
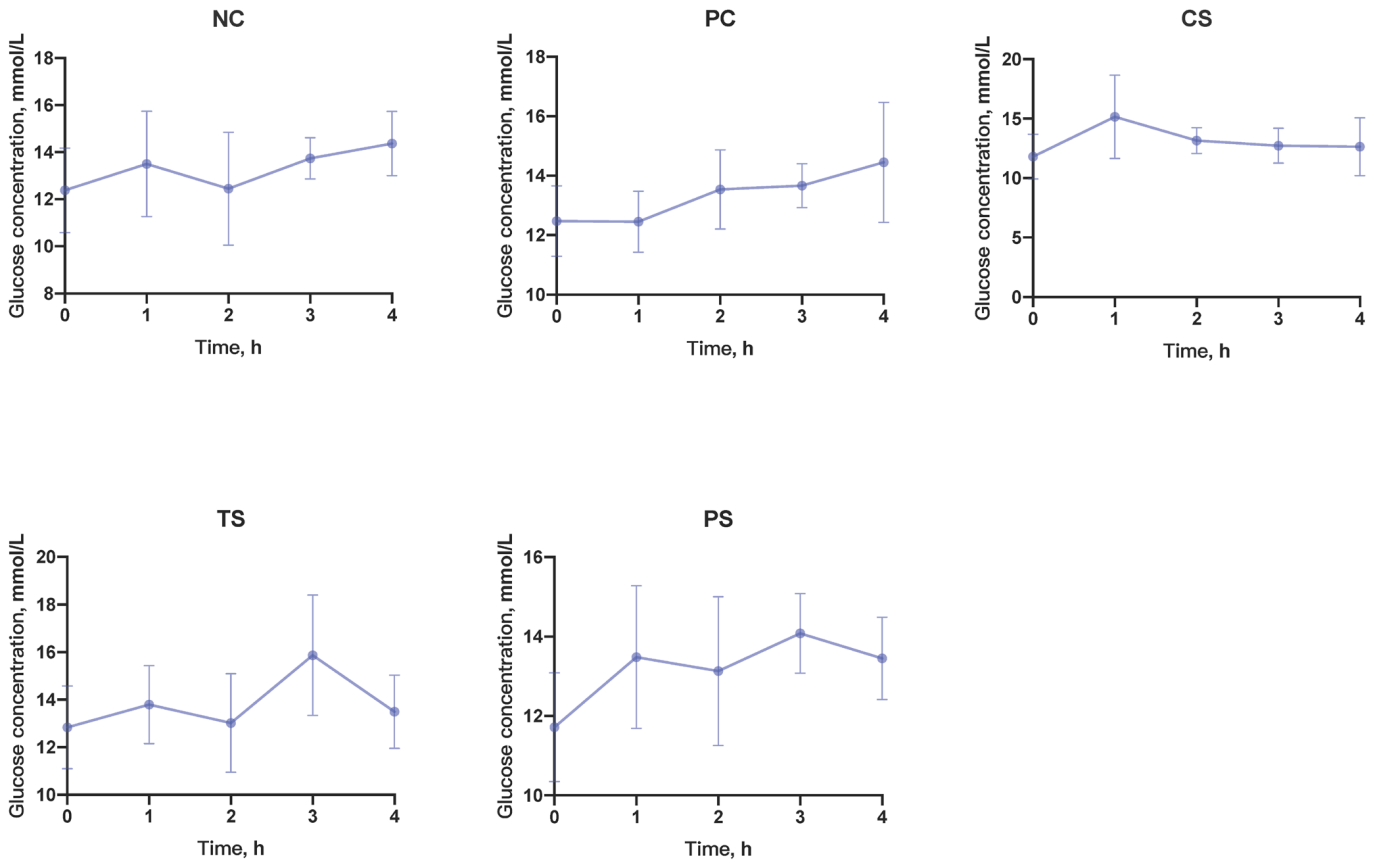
Influence of dietary treatment on postprandial blood glucose level in broiler chickens. EGCG = Epigallocatechin gallate; NC = basal corn–soybean meal diet without EGCG supplementation, PC = NC + 500 mg/kg EGCG, CS = 20% purified corn starch + corn–soybean meal + 500 mg/kg EGCG, TS = 20% cassava starch + corn–soybean meal + 500 mg/kg EGCG, PS = 20% pea starch + corn–soybean meal + 500 mg/kg EGCG (*n* = 6).

Different postprandial blood glucose response patterns were observed among the dietary treatments under EGCG supplementation (Figure 1). Blood glucose concentrations were repeatedly measured from the same birds, and the temporal response patterns are presented descriptively. Broilers fed the CS diet exhibited a transient increase in blood glucose concentration at 1 h after feeding, followed by a gradual decline toward basal levels. In contrast, the TS treatment delayed the glucose peak until 3 h postprandially, at which glucose concentration was higher than in the other groups (Table S1, *p* < 0.05). Birds receiving the PS diet maintained relatively stable glucose concentrations throughout the postprandial period.

### 3.3. Intestinal Starch Utilization and Enzyme Activities

**Figure 2:**
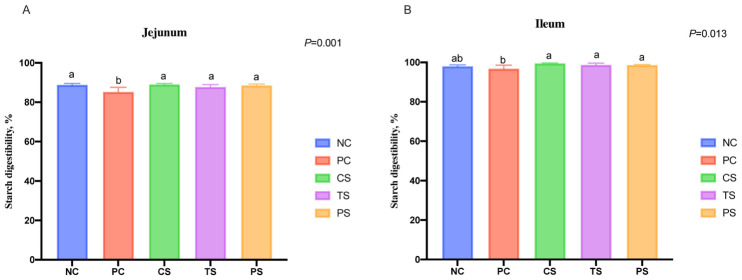
Influence of dietary treatment on starch digestibility in jejunum (**A**) and ileum (**B**). ^a,b^ Values without common superscripts in the same row differ significantly (*p* < 0.05). NC = basal corn–soybean meal diet without EGCG supplementation, PC = NC + 500 mg/kg EGCG, CS = 20% purified corn starch + corn–soybean meal + 500 mg/kg EGCG, TS = 20% cassava starch + corn–soybean meal + 500 mg/kg EGCG, PS = 20% pea starch + corn–soybean meal + 500 mg/kg EGCG (*n* = 6).

Dietary treatment affected apparent starch digestibility in the jejunum and ileum (Figure 2). In the jejunum, starch digestibility was lower in the PC group compared with the NC, CS, TS, and PS groups (*p* < 0.05). In the ileum, starch source and EGCG combination groups showed higher starch digestibility than the PC group (*p* < 0.05).

Activities of starch digestion enzymes in the jejunal mucosa are presented in Table 4. Dietary treatments did not affect α-amylase activity (*p* > 0.05). Sucrase activity was affected by dietary treatment, with the PS group showing a lower activity than the NC, PC, CS, and TS groups (*p* < 0.05). Maltase activity was highest in the TS group, which was greater than that in the PC, CS, and PS groups (*p* < 0.05), while the NC group showed an intermediate level.

### 3.4. Intestinal Energy Status

Table 5 shows the effects of dietary treatments on mucosal energy status in the jejunum and ileum. Significant differences were observed in AMP, ATP, cAMP, and the AMP/ATP ratio among treatments (*p* < 0.001).

In the jejunum, compared with the NC group, dietary supplementation with EGCG (PC, CS, TS, and PS groups) significantly decreased AMP levels, while CS and PS treatments showed higher AMP levels than TS treatment (*p* < 0.05). In contrast, ATP concentrations were significantly higher in the CS and TS groups when compared to the PS group. The AMP/ATP ratio was significantly lower in the CS and TS groups than in the other treatments (*p* < 0.05). Moreover, cAMP content was significantly elevated in both the CS and PS treatments.

In the ileum, AMP levels were generally lower than those in the jejunum. Supplementation with EGCG resulted in significantly reduced AMP levels in the cassava starch and pea starch treatments, compared to the corn starch group (*p* < 0.05). ATP concentrations were increased in the CS and TS groups compared with the NC, PC, and PS groups (*p* < 0.05). The AMP/ATP ratio was lowest in the TS group, whereas the NC group showed the highest value (*p* < 0.05). Ileal cAMP concentration was highest in the CS group, followed by the PS group, while no differences were observed among the NC, PC, and TS groups.

### 3.5. Energy Metabolism-Related Enzyme Activities in Jejunal and Ileal Mucosa

The activities of Na^+^-K^+^-ATPase, Cs, and PDH in the jejunum and ileum are presented in Table 6. Significant differences in enzyme activities were observed across treatments in both intestinal segments (*p* < 0.05).

In the jejunum, Na^+^-K^+^-ATPase activity was higher in the CS group than the other groups (*p* < 0.001). Cs activity in the CS group was comparable to that in the NC group but higher than that in the PC, TS, and PS groups (*p* < 0.001). Similarly, jejunal PDH activity did not differ between the CS and NC groups, whereas both groups showed higher values than the PC, TS, and PS groups (*p* < 0.001). In the ileum, Na^+^-K^+^-ATPase activity was higher in the PC and PS groups than in the NC, CS, and TS groups (*p* < 0.001), with no difference between the PC and PS groups. Cs activity was higher in the CS, TS, and PS groups than in the NC and PC groups (*p* < 0.001). Among the starch-containing groups, the CS group showed a higher Cs activity than the PS group, whereas the TS group did not differ from either group. Ileal PDH activity was higher in the PC group compared with the other treatments (*p* < 0.001).

### 3.6. Expression of Genes Related to Glucose Transport and Energy Metabolism

Table 7 presents the relative gene expression in the intestine of broiler chickens. Dietary treatments significantly affected the expression of SGLT1 and GLUT2 in the jejunum, as well as SGLT1 expression in the ileum (*p* < 0.05). In addition, jejunal HIF-1α and HK2 expression and ileal HK2 expression were also significantly influenced by dietary treatments (*p* < 0.05). No significant effects were observed for jejunal PDK1 expression or for ileal GLUT2, HIF-1α, and PDK1 expression (*p* > 0.05), although ileal GLUT2 expression showed a tendency toward significance (*p* = 0.066).

In the jejunum, SGLT1 expression was greater in the PC group than in the CS and TS groups (*p* < 0.05). The CS group exhibited significantly lower SGLT1 expression in the jejunum compared to the PC group (*p* < 0.05). Jejunal GLUT2 expression was upregulated in the PC group compared with all other treatments (*p* < 0.05). Similarly, jejunal HIF-1α expression was higher in the PC group than in the remaining treatments (*p* < 0.05). For HK2 expression, the PS group exhibited the highest jejunal expression level, which was greater than that of the PC, CS, and TS groups (*p* < 0.05). In the ileum, the CS group showed higher SGLT1 expression than the other treatments (*p* < 0.05). Compared with the NC group, CS and PS significantly upregulated Ileal HK2 expression (*p* < 0.05).

### 3.7. Cecal Microbial Compositions

#### 3.7.1. Bacterial α-Diversity

As shown in Table 8, significant differences were observed among treatment groups in the Chao1, Observed_features, and Pielou_e indices (*p* < 0.05), whereas no significant differences were detected in the Dominance, Goods_coverage, Shannon, or Simpson indices (*p* > 0.05). Compared with the NC and CS groups, the PS group exhibited significantly lower Chao1 and Observed_features indices (*p* < 0.05). In terms of community evenness, the Pielou_e index was significantly reduced in the PC, TS, and PS groups compared with the NC group (*p* < 0.05). No significant differences were observed among treatment groups for the Dominance, Goods_coverage, Shannon, or Simpson indices (*p* > 0.05).

#### 3.7.2. Bacterial β-Diversity and Taxonomic Composition of Microbial Communities

**Figure 3:**
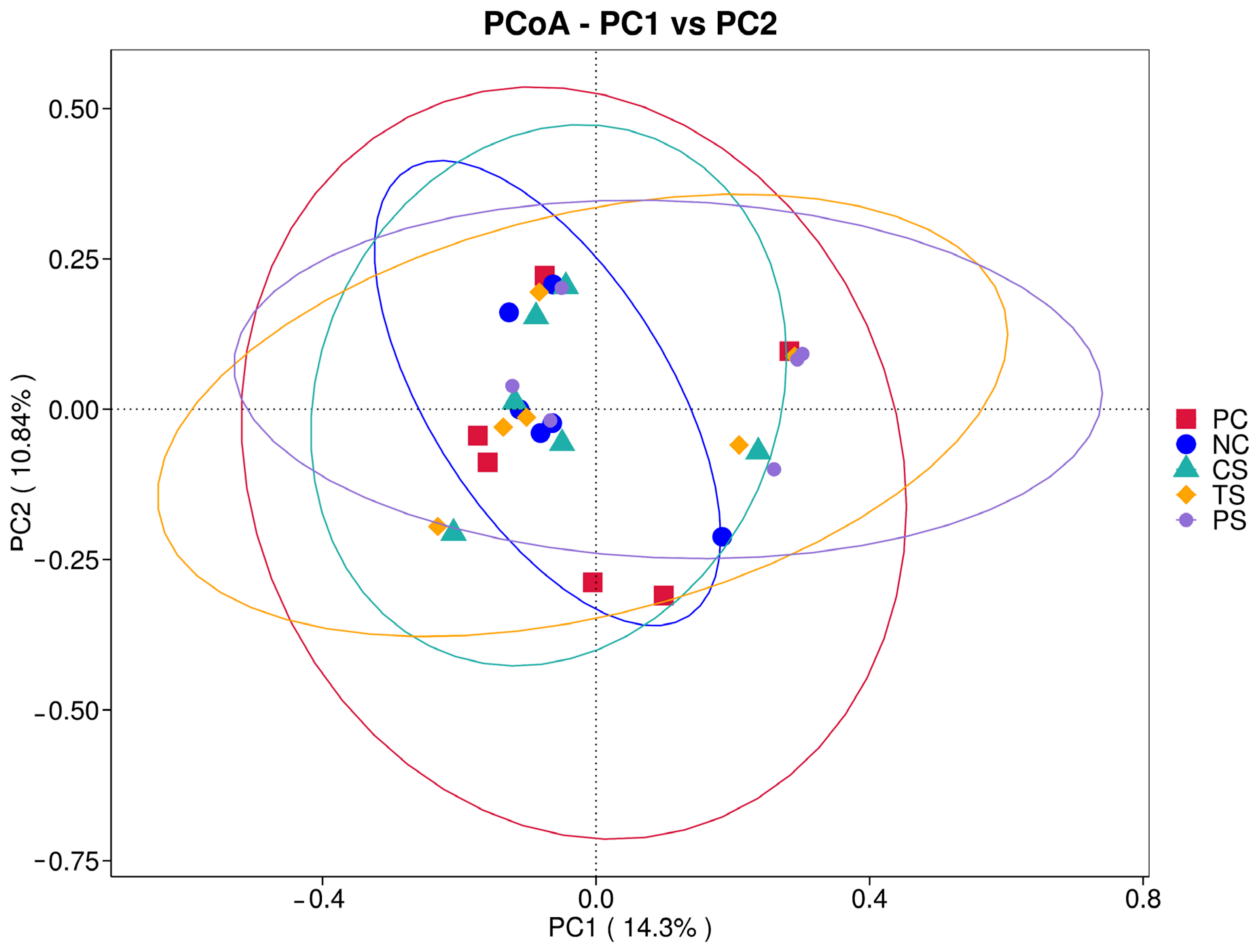
PCoA plot of the beta diversity analysis of cecal samples from birds fed with different starch supplementation with EGCG. NC = basal corn–soybean meal diet without EGCG supplementation, PC = NC + 500 mg/kg EGCG, CS = 20% purified corn starch + corn–soybean meal + 500 mg/kg EGCG, TS = 20% cassava starch + corn–soybean meal + 500 mg/kg EGCG, PS = 20% pea starch + corn–soybean meal + 500 mg/kg EGCG.

Bacterial β-diversity was assessed using principal coordinates analysis (PCoA) based on the Bray–Curtis dissimilarity index. PCoA plots showed no distinct separation among treatment groups (Figure 3). ANOSIM analysis indicated significant differences between PS and PC groups and between PS and NC groups (0.25 < R < 0.75, *p* < 0.05) (Table S2).

**Figure 4:**
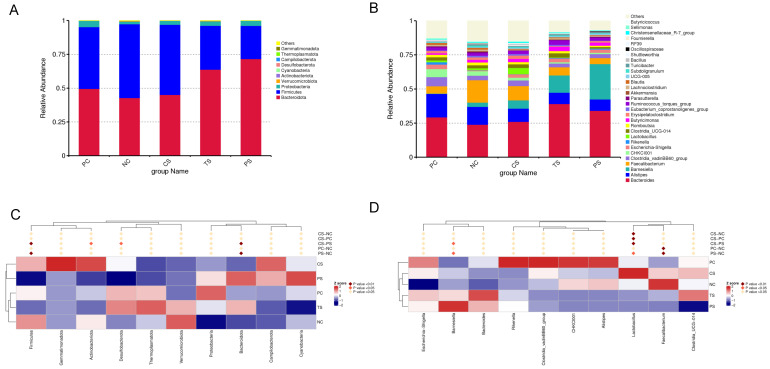
Relative top 10 abundances at the phylum (**A**) and genus level (**B**) in the birds’ cecum treated with different starch sources supplemented with EGCG. Significantly different microbiota between experimental groups at the (**C**) phylum level and (**D**) genus level. NC = basal corn–soybean meal diet without EGCG supplementation, PC = NC + 500 mg/kg EGCG, CS = 20% purified corn starch + corn–soybean meal + 500 mg/kg EGCG, TS = 20% cassava starch + corn–soybean meal + 500 mg/kg EGCG, PS = 20% pea starch + corn–soybean meal + 500 mg/kg EGCG.

The taxonomic composition of bacterial communities is shown in Figure 4. At the phylum level (Figure 4A), the dominant taxa (top 10) included Bacteroidota, Firmicutes, Proteobacteria, and Verrucomicrobiota. The relative abundance of Firmicutes in the CS and NC groups was significantly higher than that in the PS group (*p* < 0.01). Actinobacteriota and Desulfobacteriota were more abundant in the CS group than in the PS group (Figure 4C). At the genus level (Figure 4B), the most abundant taxa (top 30) included *Bacteroides*, *Alistipes*, *Barnesiella*, and *Faecalibacterium*. The relative abundance of *Lactobacillus* was significantly higher in the CS group than in the other groups. *Faecalibacterium* was more abundant in the NC group than in the PC and PS groups. *Barnesiella* abundance was higher in the PS group than in the CS and NC groups (Figure 4D).

**Figure 5:**
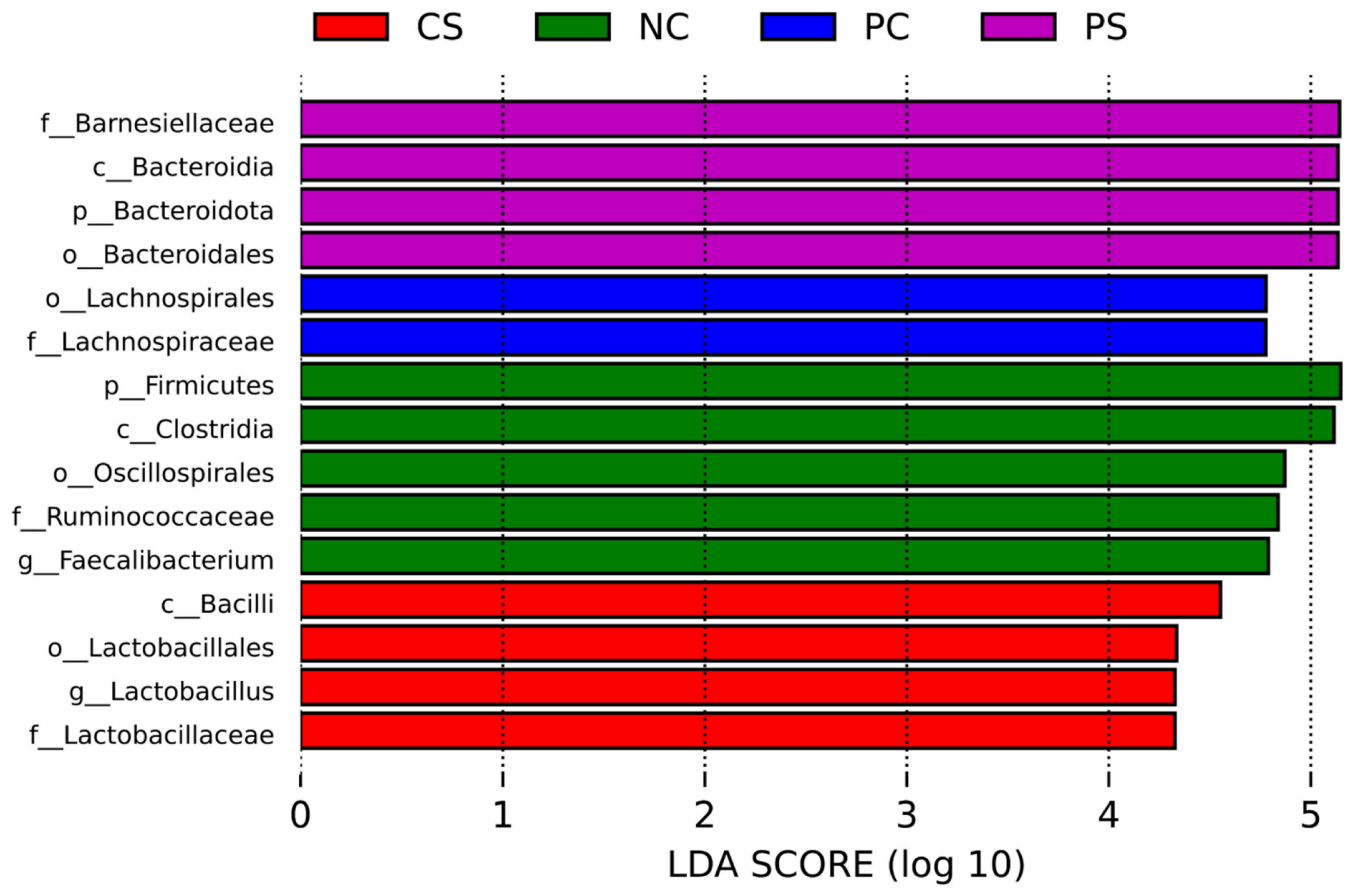
Effect size (LEfSe) analysis between experimental treatments. NC = basal corn–soybean meal diet without EGCG supplementation, PC = NC + 500 mg/kg EGCG, CS = 20% purified corn starch + corn–soybean meal + 500 mg/kg EGCG, PS = 20% pea starch + corn–soybean meal + 500 mg/kg EGCG (*n* = 6).

LEfSe analysis identified 15 discriminative features (LDA score > 2) with significant differences in relative abundance across all treatment groups (Figure 5). At the family level, the microbiota of the PS group showed enrichment of *Barnesiellaceae* from the order *Bacteroidales*, the class *Bacteroidia*, and the phylum *Bacteroidota*. The PC group exhibited significant increases in *Lachnospiraceae* and *Lachnospirales*. The NC group was significantly enriched in *Firmicutes*, *Clostridia*, *Oscillospirales*, *Ruminococcaceae*, and *Faecalibacterium*. The CS group was enriched with *Lactobacillus* from the family *Lactobacillaceae* and the order *Lactobacillales*, and showed an increase in the relative abundance of *Bacilli*. No significant differences in microbiota were observed in the TS group.

### 3.8. Analysis of Cecal Differential Metabolites Between Treatments

**Figure 6:**
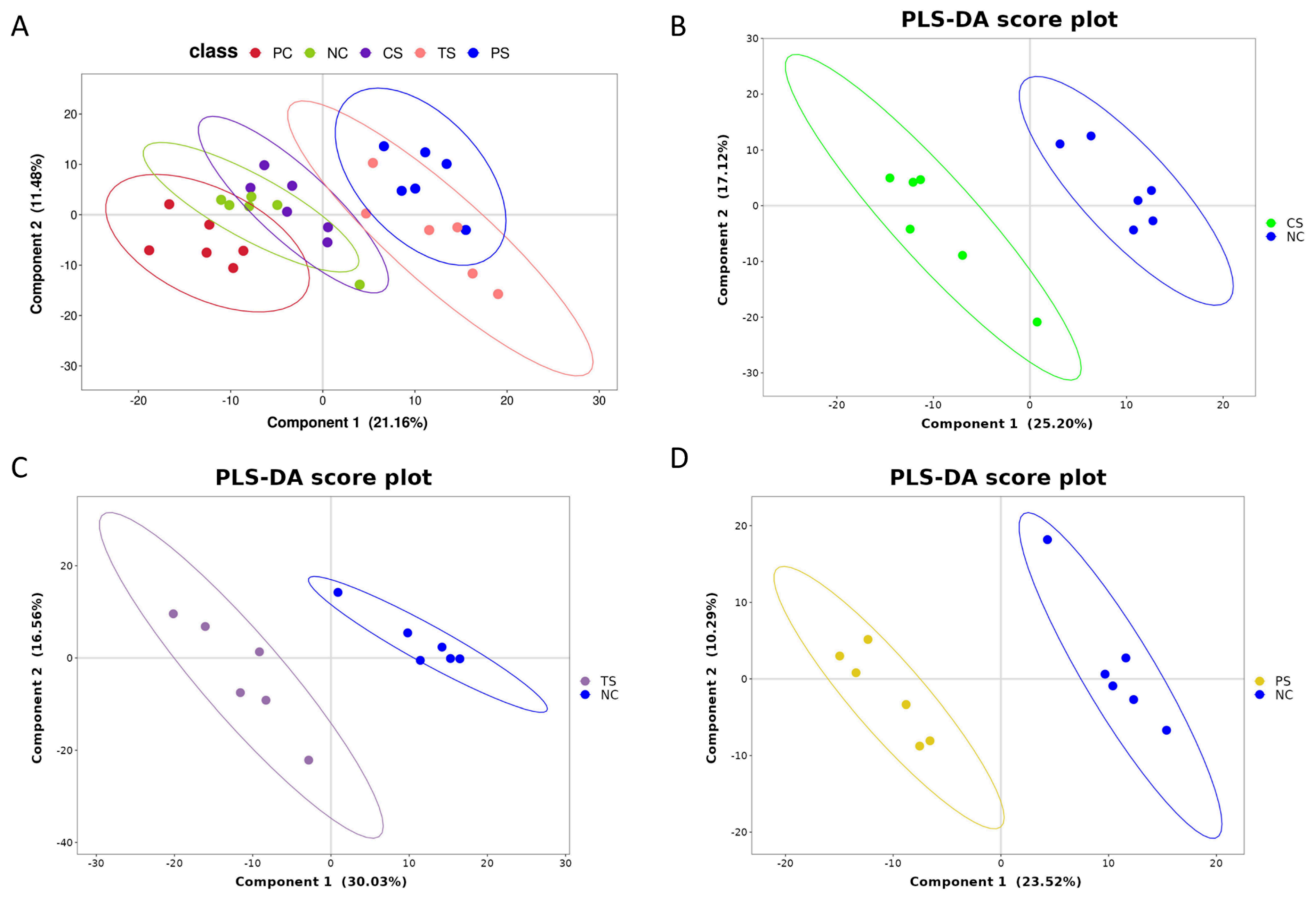
PLS-DA analysis of cecal metabolites in all experimental groups (**A**), CS and NC group (**B**), TS and NC group (**C**), and PS and NC group (**D**) of broiler chickens. NC = basal corn–soybean meal diet without EGCG supplementation, PC = NC + 500 mg/kg EGCG, CS = 20% purified corn starch + corn–soybean meal + 500 mg/kg EGCG, TS = 20% cassava starch + corn–soybean meal + 500 mg/kg EGCG, PS = 20% pea starch + corn–soybean meal + 500 mg/kg EGCG.

The PLS-DA score plots for all the experimental groups, CS vs. NC, TS vs. NC, and PS vs. NC comparisons are presented in Figure 6. Distinct clustering of samples was observed among the treatment groups, indicating clear separation and substantial differences in metabolomic profiles.

**Figure 7:**
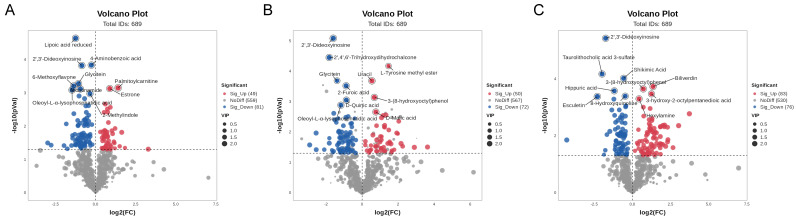
Volcano plot analysis of cecal metabolites between the CS vs NC group (**A**), TS vs NC group (**B**), and PS vs NC group (**C**). Significantly differential metabolites are shown as red (up) or blue (down) dots, whereas a gray dot represents a non-significant difference in metabolites. NC = basal corn–soybean meal diet without EGCG supplementation, CS = 20% purified corn starch + corn–soybean meal + 500 mg/kg EGCG, TS = 20% cassava starch + corn–soybean meal + 500 mg/kg EGCG, PS = 20% pea starch + corn–soybean meal + 500 mg/kg EGCG.

The differential metabolites of comparison groups were screened by combining the criteria of *p*-value < 0.05 and VIP ≥ 1 for the metabolites. As shown in Figure 7, for the CS group, volcano plot analysis identified 49 up-regulated and 81 down-regulated metabolites compared with the NC group. Metabolites related to lipid metabolism and redox processes, including lipoic acid, nicotinamide, and oleoyl-L-α-lysophosphatidic acid, were down-regulated, whereas palmitoylcarnitine and estrone were up-regulated. In the TS group, 122 metabolites were identified as significantly differentially expressed, including 50 up-regulated and 72 down-regulated metabolites compared with NC treatment. Among these, 2′,3′-dideoxyinosine, 2′,4′,6′-trihydroxydihydrochalcone, glycitein, 2-furoic acid, D-quinic acid, and oleoyl-L-α-lysophosphatidic acid were down-regulated, whereas L-tyrosine methyl ester, uracil, 3-(8-hydroxyoctyl)phenol, and D-malic acid were up-regulated. In the PS group, a total of 159 differential metabolites were detected, with 83 up-regulated and 76 down-regulated metabolites. Biliverdin, 3-(8-hydroxyoctyl)phenol, hexylamine, and 3-hydroxy-2-octylpentanedioic acid were up-regulated, whereas 2′,3′-dideoxyinosine, taurolithocholic acid 3-sulfate, shikimic acid, hippuric acid, esculetin, and 8-hydroxyquinoline were down-regulated.

### 3.9. KEGG Enrichment Analysis

The top 10 KEGG pathways enriched among the comparison groups are presented in Figure S1. In the CS vs. NC comparison, metabolites were significantly enriched in pathways related to the tricarboxylic acid (TCA) cycle, starch and sucrose metabolism, and lipoic acid metabolism. In the TS vs. NC comparison, enriched pathways were mainly associated with amino acid metabolism, including lysine degradation, alanine, aspartate, and glutamate metabolism. In contrast, the PS vs. NC comparison showed significant enrichment in the pentose phosphate pathway and the biosynthesis of unsaturated fatty acids.

### 3.10. Correlation Analysis Between Intestinal Energy Status, Cecal Microbiota, and Cecal Metabolites

**Figure 8:**
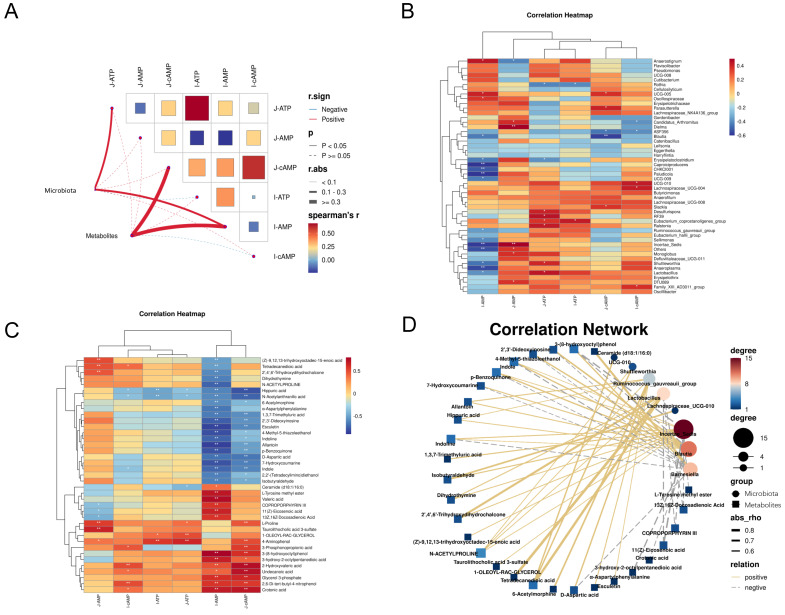
Spearman correlation analysis of cecal microbiota, metabolites, and intestinal energy status. (**A**) Integrated correlation among microbiota, metabolites, and energy parameters. (**B**) Microbiota–energy correlations. (**C**) Metabolite–energy correlations. (**D**) Microbiota–metabolite correlation network. * presented a significant difference (*p* < 0.05), ** presented an extremely significant difference (*p* < 0.01).

Spearman correlation analysis revealed significant associations among cecal microbiota, metabolites, and intestinal energy-related parameters (Figure 8). Cecal metabolites were positively correlated with jejunal cAMP levels and ileal AMP content, while cecal microbiota showed positive correlations with jejunal ATP levels and ileal AMP content (Figure 8A).

The top 20 genera significantly correlated with intestinal energy levels are shown in Figure 8B. *Lactobacillus*, *Shuttleworthia*, *Ralstonia*, *RF39*, *Desulfurispora*, and *Slackia* were positively correlated with jejunal ATP levels, while *Anaerostignum*, *UCG-005*, and *Oscillospiraceae* were positively correlated with ileal AMP content. Jejunal and ileal ATP levels were positively associated with the *Eubacterium coprostanoligenes* group and negatively correlated with *Rothia*. Jejunal ATP was also positively correlated with *Lactobacillus* and *Shuttleworthia*. Both jejunal and ileal cAMP levels were negatively associated with *ASF356*, whereas ileal AMP was positively correlated with *Anaerostipes*. As shown in Figure 8D, Valeric acid, L-tyrosine methyl ester, and crotonic acid were positively correlated with ileal AMP, while undecanoic acid, crotonic acid, and glycerol 3-phosphate were positively associated with jejunal cAMP. Hippuric acid and N-acetylanthranilic acid were negatively correlated with intestinal energy-related parameters.

Correlation network analysis revealed strong associations between specific microbial genera and metabolites (Figure 8C). *Lactobacillus*, *Blautia*, *Barnesiella*, and *Incertae sedis* were identified as key taxa with extensive connections to multiple metabolites. Meanwhile, crotonic acid and glycerol 3-phosphate exhibited strong associations with several microbial genera. In addition, polyphenol-derived metabolites, including 2′,4′,6′-trihydroxydihydrochalcone and 3-(8-hydroxyoctyl)phenol, showed close associations with *Lactobacillus* and *Barnesiella*, respectively.

## 4. Discussion

### 4.1. Growth Performance

The effects of EGCG on growth performance appeared to be dependent on dietary composition and starch characteristics [29,30,31]. In the present study, the EGCG-only group exhibited reduced growth performance, which may be attributed to the inhibitory effects of EGCG on digestive enzymes and nutrient availability [32]. However, replacing corn with purified corn starch in EGCG-supplemented diets reversed this trend, resulting in improved ADG (59.68 g/d) and feed efficiency (1.62) among all treatments (*p* < 0.05). Janaswamy [33] previously reported that native starch could serve as a carrier for plant polyphenols, facilitating the delivery of bioactive compounds and promoting gut health. In addition, a corn starch-containing diet under EGCG supplementation may modulate starch hydrolysis while maintaining glucose absorption through upregulated SGLT1 expression, which has been associated with improved growth performance. As reported by Liu et al. [34] lower concentration of amylose in the diet could improve glucose absorption and elevated growth performance. Furthermore, tea polyphenols have been reported to modify starch hydrolysis through interactions with starch molecules and digestive enzymes, particularly in starches with different amylose contents [35]. Therefore, the altered digestion characteristics of pea starch under EGCG supplementation may contribute to the differences in starch digestibility and growth performance observed in this study.

### 4.2. Starch Utilization and Glucose Availability

Starch digestion dynamics play a critical role in regulating glucose availability and metabolic efficiency. Polyphenols can form non-inclusion complexes with starch, altering enzymatic accessibility and hydrolysis rates [36]. In the present study, EGCG supplementation modulated starch digestion across different starch sources, contributing to a more stable glucose supply. Beyond digestion, EGCG has also been reported to enhance glucose utilization by mitigating insulin signaling disturbances and regulating glucolipid absorption [15]. Cassava starch, characterized by lower structural stability [37], exhibited improved glucose stability under EGCG supplementation, consistent with previous findings [38]. Similarly, the structural characteristics of pea starch, such as its high amylose content, may contribute to differential responses to EGCG supplementation by influencing starch hydrolysis and digestion kinetics [39,40,41].

Dietary treatments also affected intestinal disaccharidase activities. Polyphenols, including EGCG, are known inhibitors of intestinal α-glucosidases like maltase and sucrase [42]. Previous studies have demonstrated that EGCG reduces maltase activity in the brush border membrane by up to 40% [43,44]. In the present study, reduced enzyme activities were observed in EGCG-supplemented groups, particularly in the PS group. However, the extent of inhibition varied across starch sources. Zhuang et al. [45] suggested that EGCG stability is influenced by intestinal physicochemical conditions, while Promthong et al. [46] reported that cassava starch reduces intestinal pH, potentially weakening EGCG-mediated enzyme inhibition. The present study suggests that different starch sources modified intestinal glucose-release characteristics under EGCG supplementation, thereby changing the continuity of energy substrate supply to the intestine.

### 4.3. Intestinal Energy Metabolism

The regulation of starch digestion was closely associated with intestinal energy metabolism. A gradual glucose release provides a stable substrate for ATP generation through glycolysis and oxidative phosphorylation [47,48]. In agreement with previous studies showing that rapidly digestible starch can disrupt intestinal energy balance [5], EGCG supplementation appeared to mitigate such effects by stabilizing glucose availability. Among the dietary treatments evaluated under EGCG supplementation, the corn starch-containing diet exhibited a more balanced intestinal energy status, with higher ATP, cAMP, and Na^+^-K^+^-ATPase activity. In contrast, the PS group showed increased Na^+^-K^+^-ATPase activity but reduced ATP levels, suggesting elevated ATP turnover and localized energy demand [49]. Additionally, the increased citrate synthase activity observed in the CS group, together with cecal metabolites KEGG enrichment of the TCA cycle, suggests an enhancement of aerobic energy metabolism. Citrate synthase is a key rate-limiting enzyme of the TCA cycle and is widely considered a marker of mitochondrial oxidative capacity [50]. Compared with rapidly digestible starch, the combination of corn starch and EGCG likely provides a more stable glucose supply, thereby sustaining pyruvate production and its subsequent entry into the TCA cycle via acetyl-CoA. In addition, polyphenols have been reported to modulate central carbon metabolism, including glycolysis and the TCA cycle [51]. Differential regulation of PDH activity further suggests that EGCG influences the balance between glycolysis and oxidative phosphorylation [52].

### 4.4. Gene Expression in Intestine

Previous studies have indicated that EGCG modulates carbohydrate digestion by regulating the expression of glucose transporters such as SGLT1 and GLUT2 [43]. The upregulated SGLT1 expression observed in the jejunum of the PC and PS groups suggests that EGCG facilitates glucose transport into enterocytes, supporting peripheral glucose uptake and reducing hepatic glucose output [53]. Moreover, the higher ileal SGLT1 expression and improved energy status in the CS group indicate a potential role of EGCG in promoting glucose absorption to meet localized energy demands. HIF-1α, a key regulator of cellular response to hypoxia, was upregulated in the jejunum of the PC and PS groups, suggesting a shift toward glycolytic metabolism under lower oxygen availability [54]. This aligns with the lower PDH activity observed in these groups, as HIF-1α activation promotes glycolysis by reducing the conversion of pyruvate to acetyl-CoA, thereby limiting TCA cycle activity [55]. Conversely, the lower HIF-1α expression in the CS and TS groups, combined with higher PDH activity, indicates a shift toward oxidative phosphorylation. The higher expression of HK2 in the PS group, a key glycolytic enzyme, further underscores the role of EGCG in modulating glycolytic pathways, particularly in starch sources with higher amylose content [56]. These findings suggest that dietary starch characteristics under EGCG supplementation were associated with differences in glucose utilization through glycolytic and oxidative metabolic pathways.

### 4.5. Intestinal Microbiota Composition and Metabolites

The intestinal microbiota plays an essential role in host nutrient digestion, energy extraction, and metabolic homeostasis. As a polyphenolic compound with relatively low intestinal absorption, EGCG can reach the hindgut in substantial amounts, where it interacts with dietary components and the gut microbiota [57]. Owing to its strong binding affinity for proteins and carbohydrates, EGCG may alter nutrient availability and digestion kinetics, thereby modifying the amount and composition of fermentable substrates entering the lower intestine [58]. In addition, polyphenols are known to modulate microbial composition and activity through selective antimicrobial effects and ecological interactions [59]. In the present study, dietary EGCG supplementation in the NC and CS groups increased the relative abundance of Firmicutes while reducing Bacteroidota, indicating that EGCG supplementation altered the overall microbial community composition. This observation is generally consistent with previous reports showing that EGCG contributes to the maintenance of intestinal microbial homeostasis [60]. The CS group showed a higher relative abundance of Firmicutes, suggesting a potential shift in microbial taxa associated with carbohydrate utilization and energy metabolism [61]. By contrast, the PS group was characterized by a distinct microbial profile, including enrichment of *Barnesiella*. Given that pea starch contains a relatively high amylose content and exhibits slower digestion characteristics, it is likely to increase the flow of undigested carbohydrate to the hindgut, thereby improving microbial taxa specialized in carbohydrate fermentation [62].

The correlation analysis further indicated that cecal microbiota was positively associated with jejunal ATP levels, suggesting a close link between microbial composition and intestinal energy status. In particular, *Lactobacillus* was positively correlated with jejunal ATP. *Lactobacillus* has been widely recognized as a beneficial genus associated with improved gut function and nutrient utilization [63,64], and its enrichment in the CS group was associated with the improved intestinal energy-related indicators observed in this study. Similarly, the *Eubacterium coprostanoligenes group* was positively associated with ATP-related parameters. As this taxon has been linked to short-chain fatty acid (SCFA) production and microbial metabolic activity, its enrichment may indicate a potential microbial pathway related to intestinal energy metabolism [65]. The present study demonstrated that a corn starch-containing diet under EGCG supplementation potentially improves the gut microbiota by promoting *Lactobacillus* and bacteria associated with SCFA production, associated with improved intestinal energy status.

Cecal metabolites were positively associated with jejunal cAMP and ileal AMP, indicating that microbial metabolic outputs may be closely linked to intestinal energy regulation. Since gut microbiota extensively transform dietary polyphenols into lower-molecular-weight metabolites with distinct biological activities [66], EGCG supplementation may influence cecal metabolite profiles through interactions between dietary polyphenols and gut microbial metabolism. In this study, 3-(8-hydroxyoctyl)phenol was consistently increased under EGCG supplementation and was associated with intestinal energy-related parameters. As a phenolic lipid-like compound, its biological activities may be influenced by intestinal exposure and microbial transformation processes [67].

Among the energy-related metabolites, glycerol 3-phosphate and crotonic acid were of particular interest. Glycerol 3-phosphate is a central intermediate linking glycolysis and lipid metabolism [68], and its correlations with ileal AMP and jejunal cAMP suggest that it may reflect alterations in metabolic flux associated with dietary starch characteristics under EGCG supplementation. As reported by Madiraju et al. [69], under conditions of excess glucose and hypoxia, glycerol-3-phosphate could shunt glucose-derived carbons away from glycolysis and lipogenesis to improve energy metabolism. Both glycerol-3-phosphate and crotonic acid were increased in response to EGCG supplementation, supporting the view that EGCG altered microbial and host-associated metabolic pathways. Crotonic acid, which is linked to crotonyl-CoA formation and fatty acid β-oxidation, may represent a metabolite associated with lipid-related energy metabolism [70]. Its negative correlation with *Barnesiella*, a member of Bacteroidota, is primarily associated with carbohydrate fermentation; therefore, the inverse relationship between *Barnesiella* and crotonic acid may reflect differences in metabolic specialization, with EGCG modulating microbial metabolism from carbohydrate-oriented fermentation toward alternative pathways related to lipid-associated energy metabolism [71]. The present findings indicate that EGCG supplementation in diets containing different starch sources modulated intestinal energy status through coordinated changes in beneficial microbiota and metabolic profiles, reflecting a potential shift in microbial energy metabolism from carbohydrate fermentation toward lipid-associated energy pathways.

## 5. Conclusions

This study demonstrates that dietary starch source influenced intestinal energy metabolism-related indicators, microbial composition, and metabolite profiles in broilers receiving EGCG supplementation. Among the evaluated dietary treatments, the corn starch-containing diet exhibited a relatively favorable overall response, as indicated by improved growth performance, enhanced intestinal energy status, and alterations in microbial and metabolic profiles. These findings suggest that modifying dietary starch characteristics under EGCG supplementation may influence glucose utilization and microbial-associated metabolic responses, providing potential nutritional strategies for improving nutrient utilization efficiency in broilers.

## Figures and Tables

**Table 1:** Feed ingredients and nutrient composition of experimental diets.

Ingredients, %	NC	PC	CS	TS	PS
Corn	59.65	59.60	37.37	37.81	38.65
Soybean meal [CP, 46%]	22.80	22.80	27.45	26.80	23.818
Corn starch	0	0	20.00	0	0
Cassava starch	0	0	0	20.00	0
Pea starch	0	0	0	0	20.00
Wheat middling	3.00	3.00	0	0	0
Corn gluten meal [CP, 60%]	5.80	5.80	6.00	6.00	7.50
DL-Met	0.30	0.30	0.30	0.32	0.35
CaHPO_4_	1.70	1.70	1.78	1.77	1.80
Limestone	0.75	0.75	0.65	0.65	0.651
L-Lys	0.70	0.70	0.50	0.50	0.58
Try	0.10	0.10	0.10	0.10	0.10
Thr	0	0	0.15	0.20	0.15
NaCl	0.30	0.30	0.35	0.30	0.301
Soybean oil	3.80	3.80	4.20	4.40	5.00
Choline chloride 50%	0.10	0.10	0.10	0.10	0.10
EGCG	0	0.05	0.05	0.05	0.05
Premix ^(1)^	1.00	1.00	1.00	1.00	1.00
Total	100.00	100.00	100.00	100.00	100.00
Nutrient levels, % ^(2)^					
AME, Mcal/kg	3.07	3.07	3.05	3.06	3.04
CP	19.03	19.03	19.00	18.80	18.50
Lys	1.158	1.160	1.112	1.096	1.075
Met	0.587	0.588	0.578	0.594	0.625
Thr	0.660	0.662	0.809	0.849	0.776
Ca	0.865	0.865	0.861	0.856	0.857
Available phosphorus	0.407	0.407	0.403	0.401	0.403
Starch ^(3)^	49.3	49.3	58.5	58.0	56.3
Amylose ^(3)^	12.3	12.3	15.8	11.0	19.7
Amylopectin ^(3)^	37.0	37.0	42.7	47.0	36.6

^(1)^ The premix provided the following per kg of diet: Fe 70 mg, Cu 20 mg, Zn 70 mg, Mn 10 mg, Se 0.25 mg, I 0.2 mg, VA 10 000IU, VD 900IU, VE 50 mg, VK 2 mg, D-pantothenic acid 20 mg, VB_12_ 0.02 mg, niacin 50 mg. ^(2)^ Calculated values. ^(3)^ Measured values. NC = basal corn–soybean meal diet without EGCG supplementation, PC = NC + 500 mg/kg EGCG, CS = 20% purified corn starch + corn–soybean meal + 500 mg/kg EGCG, TS = 20% cassava starch + corn–soybean meal + 500 mg/kg EGCG, PS = 20% pea starch + corn–soybean meal + 500 mg/kg EGCG.

**Table 2:** Primers used for quantitative real-time PCR.

Gene Name	Accession Number	Forward Sequence (5′ to 3′)	Reverse Sequence (5′ to 3′)
SGLT1	XM_415247	AGATTTGGAGGGCACAGGAT	GCCCAAAGAGATTTGGATGA
GLUT2	Z22932	CCGCAGAAGGTGATAGAAGC	ATTGTCCCTGGAGGTGTT
HIF-1α	XM_046917648.1	ATCAGAGTGGTTGTCCAGCAG	CAGTCCAAGCCCACCTTACT
PDK1	NM_001031352.4	GTGGCGGAGGTGTTCCTATGAG	GTATTGTGCGTACAGGCGTGATATG
HK2	NM_204212.2	CCACCGCCTCCGTCAAGATG	CCAGGTCCAGTGCCAAGAAGTC
β-actin	NM_205518	GAGAAATTGTGCGTGACATCA	CCTGAACCTCTCATTGCCA

**Table 3:** Influence of dietary treatment on growth performance in broiler chickens.

Items	NC	PC	CS	TS	PS	SEM	*p*-Value
Body weight, g							
14 d	352.78	358.89	363.89	362.78	356.67	2.256	0.536
35 d	1563.89 ^b^	1306.67 ^a^	1617.22 ^b^	1593.33 ^b^	1596.11 ^b^	21.123	<0.001
42 d	2042.22 ^b^	1731.11 ^a^	2081.67 ^b^	2022.22 ^b^	2042.78 ^b^	22.843	<0.001
14–35 d							
ADG, g	57.67 ^a^	45.13 ^b^	59.68 ^a^	58.60 ^a^	59.02 ^a^	0.994	0.001
ADFI, g	95.16 ^a^	79.69 ^b^	96.28 ^a^	95.94 ^a^	98.14 ^a^	1.172	0.001
FCR	1.66 ^b^	1.77 ^a^	1.62 ^b^	1.64 ^b^	1.66 ^b^	0.014	0.004
35–42 d							
ADG, g	95.67	84.89	92.89	85.78	89.33	1.926	0.345
ADFI, g	176.04 ^a^	155.96 ^b^	180.58 ^a^	173.07 ^a^	179.31 ^a^	1.987	0.001
FCR	1.88	1.85	1.96	2.07	2.02	0.036	0.303
14–42 d							
ADG, g	61.89 ^a^	52.64 ^b^	63.08 ^a^	61.28 ^a^	61.90 ^a^	0.692	0.001
ADFI, g	110.14 ^a^	93.81 ^b^	111.89 ^a^	110.23 ^a^	113.17 ^a^	1.201	0.001
FCR	1.78 ^b^	1.79 ^ab^	1.78 ^b^	1.80 ^ab^	1.83 ^a^	0.007	0.125

^a,b^ Values without common superscripts in the same row differ significantly (*p* < 0.05). ADG = average daily gain; ADFI = average daily feed intake; FCR = feed conversion ratio. NC = basal corn–soybean meal diet without EGCG supplementation, PC = NC + 500 mg/kg EGCG, CS = 20% purified corn starch + corn–soybean meal + 500 mg/kg EGCG, TS = 20% cassava starch + corn–soybean meal + 500 mg/kg EGCG, PS = 20% pea starch + corn–soybean meal + 500 mg/kg EGCG.

**Table 4:** Influence of dietary treatment on starch digestion enzymes in jejunum mucosa in broiler chickens.

Treatment	NC	PC	CS	TS	PS	SEM	*p*-Value
α-amylase, U/mg prot	0.518	0.683	0.493	0.510	0.575	0.044	0.675
Sucrase, U/mg prot	204.917 ^a^	172.969 ^a^	185.545 ^a^	190.285 ^a^	99.082 ^b^	11.954	0.032
Maltase, U/mg prot	223.244 ^ab^	197.707 ^b^	152.231 ^b^	322.827 ^a^	207.840 ^b^	16.823	0.014

^a,b^ Values without common superscripts in the same row differ significantly (*p* < 0.05). NC = basal corn–soybean meal diet without EGCG supplementation, PC = NC + 500 mg/kg EGCG, CS = 20% purified corn starch + corn–soybean meal + 500 mg/kg EGCG, TS = 20% cassava starch + corn–soybean meal + 500 mg/kg EGCG, PS = 20% pea starch + corn–soybean meal + 500 mg/kg EGCG (*n* = 6).

**Table 5:** Influence of dietary treatment on intestinal mucosa energy status in broiler chickens (nmol/L).

Treatment	NC	PC	CS	TS	PS	SEM	*p*-Value
Jejunum							
AMP	286.52 ^a^	231.45 ^c^	266.48 ^b^	218.87 ^c^	259.73 ^b^	4.993	<0.001
ATP	691.06 ^b^	657.38 ^bc^	848.26 ^a^	851.66 ^a^	625.10 ^c^	18.797	<0.001
cAMP	48.55 ^b^	43.49 ^c^	57.78 ^a^	50.64 ^b^	54.88 ^a^	1.021	<0.001
AMP/ATP	0.416 ^a^	0.353 ^b^	0.314 ^c^	0.257 ^d^	0.417 ^a^	0.012	<0.001
Ileum							
AMP	258.52 ^a^	220.50 ^bc^	248.99 ^a^	214.35 ^c^	230.78 ^b^	3.547	<0.001
ATP	652.36 ^b^	638.63 ^b^	723.81 ^a^	751.27 ^a^	620.57 ^b^	11.646	<0.001
cAMP	44.26 ^c^	43.85 ^c^	50.61 ^a^	44.17 ^c^	47.18 ^b^	0.594	<0.001
AMP/ATP	0.398 ^a^	0.346 ^b^	0.345 ^b^	0.286 ^c^	0.374 ^ab^	0.008	<0.001

^a,b,c^ Values without common superscripts in the same row differ significantly (*p* < 0.05). ATP = adenosine triphosphate; cAMP = cyclic adenosine monophosphate; AMP = adenosine monophosphate. NC = basal corn–soybean meal diet without EGCG supplementation, PC = NC + 500 mg/kg EGCG, CS = 20% purified corn starch + corn–soybean meal + 500 mg/kg EGCG, TS = 20% cassava starch + corn–soybean meal + 500 mg/kg EGCG, PS = 20% pea starch + corn–soybean meal + 500 mg/kg EGCG *(n* = 6).

**Table 6:** Influence of dietary treatment on energy metabolism-related enzyme activities in the intestine of broiler chickens.

Treatment	NC	PC	CS	TS	PS	SEM	*p*-Value
Jejunum							
Na^+^-K^+^-ATPase, ng/L	419.31 ^bc^	433.12 ^b^	499.58 ^a^	402.77 ^c^	413.64 ^bc^	7.031	<0.001
Cs, pg/mL	111.55 ^a^	86.16 ^c^	110.95 ^a^	91.23 ^bc^	93.33 ^b^	2.147	<0.001
PDH, ng/L	212.59 ^a^	166.06 ^c^	215.59 ^a^	185.86 ^b^	188.94 ^b^	3.663	<0.001
Ileum							
Na^+^-K^+^-ATPase, ng/L	398.35 ^b^	497.46 ^a^	418.88 ^b^	413.51 ^b^	469.99 ^a^	8.15	<0.001
Cs, pg/mL	90.25 ^c^	87.73 ^c^	113.27 ^a^	110.89 ^ab^	106.01 ^b^	2.16	<0.001
PDH, ng/L	192.39 ^b^	209.46 ^a^	169.98 ^c^	188.23 ^b^	195.17 ^b^	2.78	<0.001

^a,b,c^ Values without common superscripts in the same row differ significantly (*p* < 0.05). Na^+^-K^+^-ATPase = sodium–potassium adenosine triphosphatase; Cs = citrate synthase; PDH = pyruvate dehydrogenase. NC = basal corn–soybean meal diet without EGCG supplementation, PC = NC + 500 mg/kg EGCG, CS = 20% purified corn starch + corn–soybean meal + 500 mg/kg EGCG, TS = 20% cassava starch + corn–soybean meal + 500 mg/kg EGCG, PS = 20% pea starch + corn–soybean meal + 500 mg/kg EGCG (*n* = 6).

**Table 7:** Influence of dietary treatment on relative gene expression in the intestine of broiler chickens.

Gene	Treatment	NC	PC	CS	TS	PS	SEM	*p*-Value
SGLT1	Jejunum	0.39 ^ab^	1.00 ^a^	0.28 ^b^	0.29 ^b^	0.89 ^ab^	0.100	0.048
	Ileum	1.00 ^b^	1.00 ^b^	1.88 ^a^	1.08 ^b^	1.33 ^b^	0.099	0.005
GLUT2	Jejunum	0.35 ^b^	1.00 ^a^	0.44 ^b^	0.53 ^b^	0.61 ^b^	0.069	0.010
	Ileum	0.35	1.00	1.78	1.76	3.62	0.333	0.066
HIF-1α	Jejunum	0.08 ^b^	1.00 ^a^	0.14 ^b^	0.06 ^b^	0.32 ^b^	0.101	0.006
	Ileum	1.30	1.00	2.50	0.95	0.77	0.248	0.242
PDK1	Jejunum	0.29	1.00	0.41	0.35	0.82	0.103	0.102
	Ileum	0.79	1.00	0.96	0.99	1.10	0.072	0.806
HK2	Jejunum	1.65 ^ab^	1.00 ^b^	0.62 ^b^	0.94 ^b^	2.18 ^a^	0.177	0.022
	Ileum	0.61 ^c^	1.00 ^bc^	1.74 ^ab^	1.27 ^bc^	2.50 ^a^	0.171	0.001

^a,b,c^ Values without common superscripts in the same row differ significantly (*p* < 0.05). NC = basal corn–soybean meal diet without EGCG supplementation, PC = NC + 500 mg/kg EGCG, CS = 20% purified corn starch + corn–soybean meal + 500 mg/kg EGCG, TS = 20% cassava starch + corn–soybean meal + 500 mg/kg EGCG, PS = 20% pea starch + corn–soybean meal + 500 mg/kg EGCG (*n* = 6).

**Table 8:** Influence of dietary treatment on α-diversity in cecal microbiota for broiler chickens.

Treatment	NC	PC	CS	TS	PS	SEM	*p*-Value
chao1	627.363 ^a^	604.068 ^ab^	686.398 ^a^	584.988 ^ab^	491.779 ^b^	20.317	0.031
dominance	0.053	0.137	0.060	0.121	0.104	0.012	0.067
goods_coverage	0.999	0.999	0.999	0.999	0.999	0.000	0.159
observed_features	608.167 ^a^	568.333 ^ab^	660.333 ^a^	561.333 ^ab^	478.333 ^b^	19.695	0.041
pielou_e	0.654 ^a^	0.556 ^c^	0.646 ^ab^	0.567 ^bc^	0.559 ^c^	0.014	0.037
shannon	6.048 ^a^	5.087 ^b^	6.052 ^a^	5.179 ^ab^	4.975 ^b^	0.154	0.031
simpson	0.947	0.863	0.940	0.879	0.897	0.012	0.067

^a,b,c^ Values without common superscripts in the same row differ significantly (*p* < 0.05). NC = basal corn–soybean meal diet without EGCG supplementation, PC = NC + 500 mg/kg EGCG, CS = 20% purified corn starch + corn–soybean meal + 500 mg/kg EGCG, TS = 20% cassava starch + corn–soybean meal + 500 mg/kg EGCG, PS = 20% pea starch + corn–soybean meal + 500 mg/kg EGCG, (*n* = 6).

## Data Availability

The 16S rRNA gene amplicon sequencing data generated in this study were submitted to the NCBI Sequence Read Archive (SRA), accession number: “PRJNA1457713”.

## Supplementary Materials

The following supporting information can be downloaded at https://www.mdpi.com/article/10.3390/ani16152445/s1: Table S1. Influence of different starch sources supplemented with EGCG on postprandial glucose levels in broiler chickens (nmol/L). Table S2. ANOSIM analysis between every two experimental treatments. Figure S1. KEGG pathway enrichment analysis of differential cecal metabolites between CS and NC groups (A), TS and NC groups (B) and PS and NC groups (C).
