# Supplementary material for: Modulation of Intestinal Energy Metabolism and Microbial Profiles by Dietary Starch Characteristics Under EGCG Supplementation in Broiler Chickens
Source: Animals (Basel). 2026 Aug 6;16(15):2445. doi: 10.3390/ani16152445 (PMC13465559; doi:10.3390/ani16152445)
Supplement: Supplementary file 1 [file animals-16-02445-s001.zip › animals-4417947-supplementary.pdf]

Table S1. Influence of different starch sources supplemented with EGCG on postprandial glucose levels in broiler chickens (nmol/L).

| Treatment | NC | PC | CS | TS | PS | SEM | *P*-value |
|---|---|---|---|---|---|---|---|
| 0 | 12.4 | 12.5 | 11.8 | 12.8 | 11.7 | 0.285 | 0.729 |
| 1 h | 13.5 | 12.5 | 15.2 | 13.8 | 13.5 | 0.407 | 0.352 |
| 2 h | 12.5 | 13.5 | 13.2 | 13.0 | 13.1 | 0.315 | 0.890 |
| 3 h | $13.7^{b}$ | $13.7^{b}$ | $12.7^{b}$ | $15.9^{a}$ | $14.1^{b}$ | 0.407 | 0.018 |
| 4 h | 14.4 | 14.5 | 12.7 | 13.5 | 13.5 | 0.322 | 0.389 |

$^{a,b}$ Values without common superscripts in the same row differ significantly ($P < 0.05$).

Table S2. ANOSIM analysis between every two experimental treatments.

| Group | R-value | *P*-value |
|---|---|---|
| PC-NC | 0.091 | 0.213 |
| PC-CS | 0.111 | 0.156 |
| PC-TS | 0.191 | 0.064 |
| PC-PS | 0.276 | 0.011 |
| NC-CS | -0.042 | 0.572 |
| NC-TS | 0.176 | 0.082 |
| NC-PS | 0.291 | 0.018 |
| CS-TS | 0.054 | 0.311 |
| CS-PS | 0.168 | 0.094 |
| TS-PS | -0.117 | 0.852 |

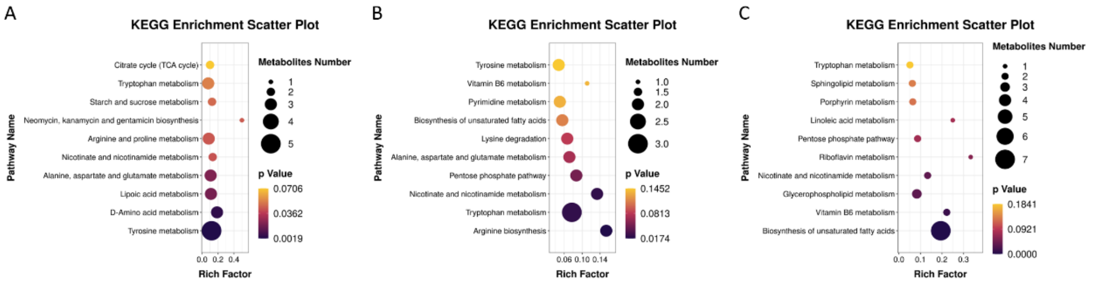


Figure S1. KEGG pathway enrichment analysis of differential cecal metabolites between CS and NC groups (A), TS and NC groups (B), and PS and NC groups (C).
